# Health Risk Assessment of Dietary Chemical Exposures: A Comprehensive Review

**DOI:** 10.3390/foods14234133

**Published:** 2025-12-02

**Authors:** Hilal Pekmezci, Simge Sipahi, Burhan Başaran

**Affiliations:** 1 Department of Elderly Care, Health Care Services Vocational School, Recep Tayyip Erdoğan University, Rize 53100, Türkiye; hilal.pekmezci@erdogan.edu.tr; 2 Department of Nutrition and Dietetics, Faculty of Health Sciences, Acibadem Mehmet Ali Aydinlar University, Istanbul 34752, Türkiye; 3 Department of Nutrition and Dietetics, Faculty of Health Sciences, Recep Tayyip Erdoğan University, Rize 53100, Türkiye

**Keywords:** food safety, chemical exposure, health risk assessment, carcinogenic, noncarcinogenic

## Abstract

Foodborne chemical exposure is a significant public health concern. Various chemical agents found in foods, including pesticide residues, heavy metals, natural toxins, compounds formed during food processing, and food additives, may result in carcinogenic or noncarcinogenic health effects in the long term. Along with discussing the main sources of dietary chemical exposure and its health impacts, this review article also covers the theoretical foundations and four main steps of chemical risk assessment. Furthermore, risk assessment approaches are investigated in the light of international organizations and guidelines, and the current debates and challenges within the field are underscored. Lastly, suggestions for safer food supply and potential future developments are presented. This comprehensive review may provide a current literature-based viewpoint on comprehending and addressing dietary chemical-associated public health issues.

## 1. Introduction

Access to safe and nutritious food is a crucial requirement for global health. In contrast, foods classified as “unsafe,” containing harmful microorganisms or chemical substances, can cause several diseases. Unwanted contaminants originating from various sources, including purposeful addition during food production, natural generation during food processing, and introduction into foods owing to environmental contamination, are the main causes of chemical exposure through diet [1,2,3]. Foodborne chemicals constitute a significant global health issue, potentially disrupting international trade [4,5]. Chemical contamination in foods poses various health hazards, ranging from acute toxicity to chronic diseases [6,7]. Chronic exposure to low doses may cause severe outcomes, including cancer, over time; thus, controlling chemical intake through diet at safe levels is essential [8,9,10].

Regulating chemical hazards in foods are among the priority areas of work for international organizations and national authorities. The Codex Alimentarius Commission, formed through the collaboration of the World Health Organization (WHO) and the Food and Agriculture Organization of the United Nations (FAO), develops scientific risk assessments associated with safeguarding consumer health and promoting equitable trade practices [11]. Risk assessment encompasses the systematic analysis of potential adverse effects of hazards on human health based on scientific data. In particular, dietary chemical risk assessment aims to quantitatively and qualitatively evaluate the potential harmful effects of a specific chemical ingested through food on human health. This process necessitates diverse approaches for chemicals that demonstrate carcinogenic properties compared with those that exhibit other toxicological effects, including neurotoxicity or endocrine disruption. This difference affects the methods and criteria employed in risk assessment.

In this review, the theoretical framework and methodologies for assessing dietary chemical exposure-associated health risks will be comprehensively addressed. First, the fundamental steps and concepts of risk assessment will be outlined, followed by a thorough investigation of the processes involved in the quantitative evaluation of exposure and its translation into health risk. This study will explore significant foodborne chemical hazards and their exposure pathways, while summarizing the guidelines and approaches established by international authorities on the topic. The final section will present the policy recommendations, research gaps, monitoring strategies, and future perspectives. Therefore, this review aims to offer a comprehensive perspective based on the general scientific literature.

## 2. Literature Search and Selection

This review was conducted as a narrative synthesis rather than a systematic review. Literature was identified through searches in PubMed, Web of Science, Scopus, and Google Scholar, complemented by grey literature sources and official guidance documents from EFSA (European Food Safety Authority), WHO/FAO, IARC (International Agency for Research on Cancer), and other relevant international bodies. The primary search window covered publications from approximately 2010 to 2025, while earlier foundational studies were included when necessary to clarify toxicological concepts. A broad set of keywords and combinations was used, including: “dietary chemical exposure”, “food contaminants”, “health risk assessment”, “benchmark dose”, “margin of exposure”, “target hazard quotient”, “hazard index”, “carcinogenic Risk”, “cumulative risk”, “mixture toxicity”, “cocktail effect”, “pesticides”, “veterinary drugs”, “mycotoxins”, “heavy metals”, “process contaminants”, “environmental pollutants”, “endocrine disruptors”, and “biomonitoring”. Selection focused on original studies evaluating dietary exposure or human health risk, high-quality reviews, major international assessments, and reports introducing emerging contaminants or novel methodological approaches. Selection focused on original studies assessing dietary exposure or human health risk, major international reports, and recent high-quality reviews representing the current state of the field. As a narrative review, no formal inclusion or exclusion criteria were applied, but emphasis was placed on relevance, scientific rigor, and representativeness.

## 3. Chemical Risk Assessment Process

Chemicals can enter the food chain via several pathways. Pesticides and veterinary drugs used in agricultural production may leave residues in food products, whereas environmental pollutants originating from industrial activities can contaminate plants and animals via air, water, and soil pathways. Furthermore, during food processing, undesirable reactive by-products may form, and chemical migration from packaging materials into food may occur [12,13,14,15,16]. In such cases, the oral ingestion of contaminated foods is the main pathway for human exposure.

**Figure 1:**
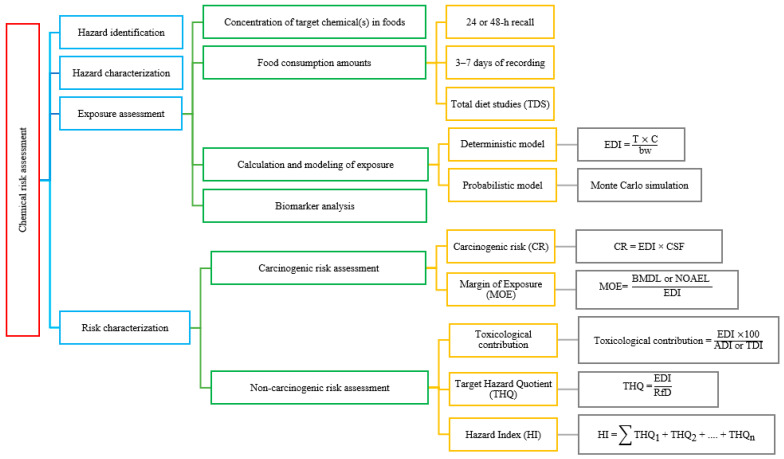
Schematic overview of the chemical risk-assessment process, illustrating the sequential stages from hazard identification to risk characterization. EDI: Estimated daily intake, T: Food consumption amount, C: Level of relevant contaminant in food, bw: Body weight, CSF Cancer slope factor, BMDL: Benchmark dose lower confidence limit, NOAEL: No observed adverse effect level, ADI: Acceptable daily intake, TDI: Tolerable daily intake, RfD: Oral reference dose.

Risk assessment in food safety is a scientific process generally conducted in four fundamental steps. These steps offer an organized framework for identifying and characterizing the potential adverse effects of chemical agents in foods on human health [17]. Figure 1 provides a schematic representation of the four main stages of chemical risk assessment—hazard identification, hazard characterization, exposure assessment, and risk characterization—and illustrates how exposure estimates are integrated to evaluate potential health risks.

### 3.1. Hazard Identification

Identifying the potential adverse health effects associated with the chemical under investigation represents the first step (Figure 1). Classifying a substance as a “hazard” depends on evidence that the agent can cause direct harmful effects in an organism or population [17,18]. All available toxicity data from human and animal studies are compiled and assessed at this stage. For instance, epidemiological studies demonstrating an association between exposure to a particular food chemical and a higher cancer incidence classify chemicals as a carcinogenic hazard. Accordingly, IARC categorizes various chemicals and exposures according to their carcinogenic potential, thereby contributing to hazard identification [19]. The hazard identification process encompasses assessing carcinogenicity alongside other toxic effects, including neurotoxicity, reproductive toxicity, and immunotoxicity. To clarify the potential harmful effects and mechanisms of action of a chemical, animal experiments, in vitro toxicity tests, and, when necessary, structure–activity relationship approaches are employed.

### 3.2. Hazard Characterization (Dose–Response Assessment)

Upon identifying the hazard, the subsequent step involves quantitatively assessing the dose or concentration at which the chemical generates the adverse effect (Figure 1) [17]. Dose–response assessment seeks to define the correlation between increasing exposure level and intensity frequency of adverse effects. At this stage, data obtained from animal studies are crucial; for example, by investigating whether a chemical administered at different doses induces liver damage or tumor formation, parameters, including the No-Observed-Adverse-Effect Level (NOAEL) or Benchmark Dose (BMD), are determined. Subsequently, safe exposure levels for humans are established. Acceptable exposure limits for noncarcinogenic effects, including the Acceptable Daily Intake (ADI) or Tolerable Daily Intake (TDI), are defined to indicate levels that are believed to pose no significant health risks. Values are frequently obtained by applying suitable safety factors, often 100-fold, to the NOAEL determined from animal studies [17]. Conversely, genotoxic carcinogens are not assigned classical ADI or TDI values as it is theoretically accepted that no dose exists that poses zero risk for these substances. In such circumstances, approaches, including the Margin of Exposure (MOE) or quantitative models for estimating cancer potency, are utilized in risk assessment [20]. The most frequently used terms in hazard characterization are defined in Table 1. Dose–response assessment is gaining significance in evaluating subthreshold effects, including the potential impacts of endocrine disruptors at minimal doses. Evidence indicates that certain chemicals may have biological effects even at low-exposure levels, challenging conventional toxicological principles [21,22]. This finding introduces uncertainty into the risk assessment process and has generated ongoing debate within the scientific community.

### 3.3. Exposure Assessment

The third step in risk assessment, exposure assessment, encompasses evaluating the degree of exposure of the target population to the chemical in question (Figure 1). Dietary exposure assessment involves integrating food consumption data with the concentration data of chemicals present in foods [17]. For this purpose, the daily intake is calculated by multiplying the quantity of food consumed by individuals within a population by the average or percentile concentration of the chemical detected in that food [23]. To facilitate comparison among various age and sex groups, exposure estimates are frequently normalized to body weight. Chronic exposure assessment is based on long-term average consumption and mean chemical levels, whereas acute exposure assessment focuses on the maximum dose that may be ingested in a single meal or within 1 day [17]. For example, in the case of pesticides, acute poisoning risk assessment can be conducted by comparing the 95th percentile of single-meal consumption values to the Acute Reference Dose (ARfD).

Exposure assessment may be performed for the general population and specific subgroups, including infants, children, and pregnant women, who are considered more vulnerable. Infants and children have higher food consumption relative to their body weight and demonstrate greater metabolic sensitivity, causing higher or more critical exposure levels than adults [24,25,26]. Furthermore, considering that dietary patterns vary across various geographical regions and cultural contexts, using actual and locally representative data in exposure assessments is recommended [17]. Recently, organizations, including EFSA, have developed extensive databases that compile food consumption patterns of European populations and have developed online tools (e.g., the EFSA Comprehensive Database, DietEx) to assist in chemical exposure calculations [27,28].

Dietary chemical exposure assessment is generally based on three main components: chemical concentration in foods, food consumption data, and calculation and modeling of exposure.

#### 3.3.1. Chemical Concentration in Food Products

By routinely monitoring programs by food control authorities, total diet studies (TDS), or from scientific literature, measured or estimated levels of the chemical under investigation in various foods can be obtained. Initial data on chemical concentration levels in foods are frequently generated by analyzing selected food samples using the laboratory capabilities of researchers. Alternatively, data from regular monitoring and inspection programs that report measured chemical concentrations in various food products can be used. Without adequate data, legally established Maximum Residue Limits (MRLs) or tolerance levels may be utilized in exposure calculations as a conservative estimate. The TDS approach functions as a method for determining reference concentrations of chemicals in food products. This approach encompasses grouping similar food items to establish composite samples, which are subsequently analyzed. Researchers utilize the resulting chemical concentration data for estimating exposure [17]. At this stage, the representativeness of analytical data is critical as methodological variations across datasets from different countries or regions may introduce uncertainty into the assessment.

#### 3.3.2. Food Consumption Data

##### Common Dietary Survey Techniques

The data regarding the quantities of various foods consumed represent the second major component of exposure calculations (Figure 1). Food consumption data may be attained through community-based, household-based, or individual-based methodologies [17,29]. To achieve this, national food consumption surveys accurately representing the dietary habits of the population are the most reliable sources [17]. Dietary surveys, food frequency questionnaires, and household budget studies are employed for collecting data on the types and quantities of foods consumed by individuals. In various countries, these surveys are conducted periodically employing frequently used methods, including “24-h recall,” “48-h recall,” or “3–7-day food record.” The 24/48-h recall method requires participants to detail all foods and beverages consumed from waking up to bedtime over the past 1 or 2 days, including information on portion size, ingredients, preparation methods, and brand. The 3–7-day food record method involves individuals documenting all foods and beverages consumed daily, along with relevant details, in a structured form [30]. Without direct consumption data, per capita average food intake may be estimated using production and supply statistics [17,29]. Nevertheless, such “food balance” approaches fail to account for variations in age, sex, or dietary culture across the population, potentially causing heightened uncertainty in exposure estimates.

##### Population-Based Databases and Screening Tools

Screening tools developed by multiple institutions for evaluating general dietary behaviors within populations may be valuable for this objective. The EFSA has standardized over 30 national dietary surveys from 22 member states to develop the Comprehensive European Food Consumption Database. This database offers comprehensive food consumption distributions across diverse age and demographic groups, ranging from infants to older adults, facilitating precise exposure calculations at the European level [27,28]. The Global Environment Monitoring System/Food database, developed by the WHO/FAO, offers food consumption patterns (cluster diets) for several geographical regions and countries. This resource enables applying regional dietary assumptions in international risk assessments in the absence of country-specific data [31].

##### Total Diet Study (TDS)

The TDS method is a comprehensive approach for assessing food consumption levels. It aims to evaluate the chronic exposure levels of the population by highlighting long-term and habitual dietary patterns rather than short-term consumption habits [17,29]. The primary strength of the TDS method is its capacity to represent chemical intakes over extended periods, unaffected by short-term fluctuations [32]. This approach is frequently employed in long-term risk assessments for compounds, including pesticide residues, heavy metals, food additives, and environmental contaminants [33,34,35]. However, it is inappropriate for assessing acute exposure, defined as single high-level intakes. The TDS can be adapted to different age, sex, and socioeconomic demographics, providing crucial public health insights by indicating whether exposure levels are increased in vulnerable groups, including infants and children, compared with adults [29].

The TDS process initiates with selecting food samples that accurately reflect the dietary habits of the population. The samples are derived from consumption data collected through national or regional dietary surveys and are intended to reflect typical eating patterns. Foods are either cooked, boiled, or fried to replicate realistic consumption conditions and are frequently combined into composite samples for analysis. Chemical substance and contaminant concentrations are measured through representative composite samples, offering an estimate of the average composition of the daily diet. This method reduces variability due to individual differences and facilitates more accurate exposure estimates at the population level. The TDS is a crucial method for food safety risk assessments and formulating public health policies. Furthermore, it is significantly involved in informing global food safety strategies through cross-country comparisons.

#### 3.3.3. Calculation and Modeling of Exposure

##### Deterministic (Screening-Level) Exposure Assessment

To assess the dietary intake of a chemical, integrating the concentration and consumption data previously outlined is necessary. This is accomplished by correlating food consumption data with chemical analysis through a standardized food classification coding system (Figure 1). Exposure assessments are typically performed through a systematic stepwise methodology. In the first step, a simple and conservative screening-level calculation is conducted, frequently employing a deterministic model. This method is utilized in screening-level and higher-tier evaluations. Deterministic modeling employs point values for exposure estimation, causing a singular point estimate as output. Furthermore, actual consumption data are frequently applied, facilitating clear and precise interpretation of the results. The deterministic model is simpler to apply than other modeling approaches, accounting for its preference among several researchers [17,29,30,36]. A more refined approach, including probabilistic modeling, may be utilized when the values obtained at this stage are alarming.

##### Probabilistic Exposure Assessment

In probabilistic modeling, chemical concentrations in foods and individual consumption amounts are analyzed as statistical distributions. Monte Carlo and analogous simulation techniques produce several random scenarios to develop a probability distribution of exposure within the population. Probabilistic assessments reflect the natural variability in the data and quantify uncertainty, providing exposure levels along with corresponding confidence intervals across various percentiles (e.g., 95th percentile) [17,29]. Unlike deterministic models that rely on single-point estimates, Monte Carlo methods generate exposure distributions by repeatedly sampling from probability distributions of food consumption and contaminant levels. The EFSA supports the more routine application of probabilistic methods when appropriate, particularly in domains like cumulative risk assessment, where these approaches are significant [37]. Specialized expertise is required in reporting the methodology and results of probabilistic modeling. These models are commonly employed for advanced evaluations and incorporate more sophisticated modeling techniques than deterministic models.

##### Biomarker-Based Exposure Assessment

Biomarker analysis, which relies on the direct quantification of chemical concentrations within the human body, is another method employed for dietary chemical exposure evaluation (Figure 1) [17,38,39]. Identifying chemicals or their metabolites in biological samples, including blood, urine, breast milk, or hair, shows the overall internal exposure of an individual. Blood lead concentration reveals lead accumulation in the body, whereas urinary mercury or pesticide metabolite levels function as biological indicators of exposure to these substances. As biomarker measurement accurately reflects true internal exposure rather than calculated intake, it offers a distinct advantage over estimates based on food consumption and food analysis. The direct representation of the total absorbed dose offers more precise data regarding the internal burden that may cause health outcomes. This method is especially useful when dietary records lack reliability or when individuals encounter chemicals from various sources, including nondietary ones, as biomonitoring can consolidate all exposure pathways into a unified measure [38]. However, several significant limitations in biomarker-based assessments exist [17,38,39]. Translating the chemical level measured in a biological fluid or tissue into a quantitative health risk is not always straightforward. As human studies rarely incorporate controlled dose data, establishing a dose–response relationship from these values is frequently difficult. Biomarkers fail to distinguish between sources of exposure. A pesticide metabolite detected in blood does not specify the particular food source or the extent of exposure; it may also represent environmental exposure or the combined effects of various foods. Biomarker data are frequently assessed together with dietary exposure models to perform source attribution analyses. A third limitation is that certain biomarkers possess short biological half-lives, indicating that they represent only recent exposure (e.g., within the past 24–48 h). This scenario complicates the inference of long-term average intake. A pesticide metabolite detected in urine suggests recent consumption of fruit with increased pesticide residue levels; however, it cannot ascertain whether this reflects a consistent dietary pattern based on a solitary measurement. Overall, biological monitoring acts as a valuable tool; nonetheless, it necessitates careful interpretation. Risk assessors frequently use biomarker data alongside conventional dietary exposure estimates to provide supporting evidence for risk characterization [40,41,42,43].

##### Estimated Daily Intake (EDI)

The result of exposure assessment is frequently an Estimated Daily Intake (EDI) value, quantified in ng, µg, or mg per kilogram of body weight per day. This value represents the quantity of a chemical consumed based on current dietary habits. This value is subsequently compared with toxicological threshold values for assessing potential health risks. Errors in dietary reporting, insufficient sampling, and dynamic changes in chemical concentrations throughout food processing can cause uncertainties in exposure assessment [17,29,37]. Dietary chemical exposure studies reveal that, for most consumers, exposure levels typically remain beneath the established safe intake limits, whereas certain subgroups with elevated consumption of specific foods or unique circumstances (e.g., contaminants in infant formulas) may encounter exceptional risk scenarios [14,25,43,44]. This result underscores that exposure assessment is a dynamic process demanding continuous updates alongside the ongoing monitoring of chemical risks in food.

### 3.4. Risk Characterization

Integrating hazard information pertaining to a specific chemical, including dose–response relationships, with exposure levels to quantitatively or qualitatively articulate the potential risk to human health, is the concluding phase of risk assessment (Figure 1) [17,29]. The risk characterization phase encompasses determining the magnitude and likelihood of adverse effects that a particular chemical exposure level may exert on a human population. Risk characterization aims to address the question: “To what extent is this population at risk from the current exposure level to this chemical?”. This assessment requires distinct methodologies for evaluating carcinogenic and noncarcinogenic effects (Figure 1).

#### 3.4.1. Carcinogenic Risk (CR) Assessment

The scientific consensus indicates that for carcinogenic chemicals, especially those with a genotoxic mechanism that causes DNA mutations, no exposure level that ensures zero risk is noted [45,46]. Consequently, the potential increase in cancer cases generally quantifies CR. The U.S. Environmental Protection Agency identifies a dose associated with a maximum of one additional cancer case per one million individuals over a lifetime as the target level for risk management [47]. CR calculations utilize cancer (oral) slope factor (CSF) values obtained from high-dose animal studies, which are subsequently extrapolated to the low-dose range through a linear model [48]. This approach involves identifying the lowest dose that induces liver tumors in mice, estimating the corresponding human-equivalent dose, and applying conservative safety margins for deriving a human risk estimate. The key question is: “To what extent does the current exposure level to this chemical increase lifetime cancer risk?”. Another method employed in the risk assessment of carcinogenic chemicals is the MOE approach (Figure 1) [29].

Although the role of the IARC mainly focuses on hazard identification, its classifications are fundamental for risk characterization. For example, in 2015, the IARC categorized processed meat products, including sausages and nitrite-containing cured meat, as Group 1 carcinogens, demonstrating that they are carcinogenic to humans. The organization reported strong evidence linking high consumption of these products to colorectal cancer [49]. Following this classification, the WHO and other health authorities offered risk management recommendations based on population-level exposure to these food types [50]. A global analysis has reported approximately 34,000 annual deaths from processed meat consumption-associated colorectal cancer [49]. This quantitative estimate illustrates the significance of CR characterization in relation to public health and dietary exposures.

#### 3.4.2. Non-CR Assessment

Regarding toxic effects aside from cancer, the threshold concept frequently determines the risk. It is assumed that no harmful effects occur below a certain dose for these chemicals. Therefore, risk characterization involves comparing exposure levels with established threshold values, including the ADI or TDI. The predominant method in exposure assessments includes calculating the toxicological contribution by determining the ratio of EDI to the relevant TDI, ADI, or provisional tolerable daily/weekly/monthly intake (PTDI/PTWI/PTMI) values [51,52,53]. The outcome is expressed as a percentage; values ≥100% indicate a potential health risk. Another commonly used method is the calculation of the Target Hazard Quotient (THQ). Exposure is considered acceptable when the THQ is ≤1; conversely, a THQ of >1 indicates a potential risk. The Hazard Index (HI) is calculated by summing the individual hazard quotients when multiple chemicals exert combined effects. When multiple chemicals affect the same organ or physiological system, their individual THQ values are added for calculating the overall risk index. For example, the THQ values of several organophosphate pesticides in food can be summed to derive a cholinesterase inhibition-associated cumulative risk value. The reference interpretation for HI values aligns with that of the THQ (Figure 1) [54,55].

In characterizing risk, the interpretation of uncertainties is also reported along with numerical findings. Factors, including the cumulative impacts of chemical mixtures, variability in individual sensitivity, and existing data gaps, are highlighted, suggesting that results necessitate careful interpretation. Nevertheless, risk characterization ultimately provides decision-makers an informed perspective. It determines whether the current dietary intake level of the chemical under investigation is acceptable for human health. Risk managers formulate suitable intervention strategies (e.g., lowering legal limits, imposing product restrictions, or issuing consumer warnings) when the risk is unacceptable. Therefore, risk characterization is a crucial step that translates scientific evidence into practical decisions for protecting public health.

## 4. Chemicals of Interest in Dietary Exposure Studies

Humans are exposed to a wide range of chemicals through their diet. Certain substances are inherently detected in foods or originate from environmental contamination, whereas others are intentionally added as food additives or generated during food processing. Dietary chemical-associated health risks are primarily determined by the specific compound and its toxicological properties. In this review, the contaminant groups were selected based on their frequent occurrence in foods, their prominence in recent dietary exposure and risk-assessment literature, and their regulatory relevance as chemicals identified by WHO/FAO, EFSA, and national authorities as priorities for public-health protection.

Table 2 presents a summary of significant chemicals of concern in dietary exposures, including environmental and agricultural contaminants, their primary food sources, origins of contamination, and associated health effects.

The significant diversity of chemicals to which humans are exposed through diet is shown in Table 2. Pesticide residues mainly arise from agricultural chemicals applied in crop production and can persist in products, including fruits, vegetables, and cereals. Several countries have set legal MRLs and follow ADI value-informed risk management strategies. In standard practice, dietary exposure estimates below the ADI for a specific pesticide are considered safe. Pesticide-related risk analyses typically evaluate carcinogenic and noncarcinogenic health risks through various indices, including the THQ, HI, and MOE. High-dose pesticide exposure may cause acute poisoning, whereas long-term low-dose exposure has been associated with heightened risks of cancer and endocrine disruption for specific compounds. The IARC has classified several broadly employed pesticides on the basis of their carcinogenic potential. Pesticide exposure is particularly crucial for children considering that their food intake relative to body weight exceeds that of adults, causing proportionally greater exposure to residues from identical foods; therefore, in pesticide risk assessments, providing special consideration to the exposure of infants and children is essential, and safety factors should be adjusted accordingly. In general, the levels of pesticide residues frequently consumed through diet are minimal, typically falling below the established ADI values based on current assessments, suggesting that the associated health risk is negligible. However, in circumstances of limited safety margins and uncertainties are present, using the pesticide in question may be restricted or banned. In Europe, large-scale monitoring programs have revealed that pesticide residues generally remain within regulatory limits; however, the potential “cocktail effect,” which is the simultaneous presence of multiple pesticides in a single product, warrants consideration [99]. The “cocktail effect” refers to the combined toxicological impact of multiple chemical substances when present together, where their mixture may lead to additive, synergistic, or antagonistic effects that differ from the toxicity of each chemical evaluated individually [99].

Potentially toxic metals may either be naturally present in the environment or infiltrate the food chain owing to industrial pollution. Lead (Pb), arsenic (As), cadmium (Cd), mercury (Hg), aluminum (Al), chromium (Cr), nickel (Ni), copper (Cu), cobalt (Co), and manganese (Mn) are frequently investigated potentially toxic metals in foods. These metals ingested via contaminated food show a tendency to bioaccumulate in the human body, raising significant concerns regarding chronic health risks caused by prolonged low-dose exposure. International authorities classify As, Pb, Cd, and Hg as priority contaminants [100]. Accordingly, to detect and mitigate the presence of potentially toxic metals in foods, several organizations and countries have developed routine monitoring and surveillance programs, aiming to maintain dietary exposure levels as low as reasonably achievable [101,102,103]. Exposures to these metals are regularly monitored in both the general population and sensitive subgroups. In general, risk assessments are performed by comparing intake levels with reference values, including the ADI, TDI, PTDI, PTWI, or PTMI. Threshold values for the projected daily consumption of various potentially hazardous metals have been established in this context [104,105]. Moreover, for metals with defined RfD and CSF values, health risk assessments for both carcinogenic and noncarcinogenic effects are frequently reported in scientific literature.

Owing to mold growth occurring under inadequate storage conditions or adverse climatic factors, mycotoxins can contaminate foods, particularly cereal grains, nuts, legumes, and fruits. Mycotoxins are undesirable contaminants that present potential health risks, leading several countries to set strict tolerance limits in food standards [106,107,108]. Geographical and climatic factors significantly impact mycotoxin exposure. The global distribution of mycotoxin-producing molds and contamination patterns in crops are altering owing to climate change [109,110]. This shift can enhance human exposure to specific mycotoxins and raise food safety concerns. Indeed, European-based studies have suggested that changing climate conditions can cause an increased frequency and concentration of toxins, such as deoxynivalenol (DON) in cereals grains, likely raising public health concerns [111,112,113]. Effective mycotoxin contamination management necessitates adopting good storage practices, alongside appropriate drying and sorting techniques, as essential preventive strategies. In certain cases, risk management strategies, including destroying highly contaminated products, are performed [114,115]. Dietary mycotoxin exposure assessments have extensively reported both CR evaluations (e.g., CR and MOE) and numerous studies on noncarcinogenic health risk assessments in the literature.

Table 3 presents a summary of significant chemicals of concern in dietary exposures, including processing, additive, and packaging contaminants, their primary food sources, origins of contamination, and associated health effects.

Thermal process contaminants generated during food preparation have integrated into the modern diet owing to contemporary lifestyles. Cooking methods, including frying, baking, and smoking, whether domestically or industrially applied, create the intended flavor and appearance while also facilitating the production of undesirable reaction products, including HMF, acrylamide, HCAs, chloropropanols, and PAHs [164,165]. HMF and acrylamide are produced in sugary and carbohydrate-rich foods, respectively. Meat cooked at high temperatures generate HCAs. Chloropropanols arise during oil refining processes, whereas PAHs are produced in foods that are directly exposed to fire or smoke. Several researchers have underscores that, owing to their potential toxic and carcinogenic effects, these compounds entail close monitoring and should be considered a critical aspect of food safety policies [166,167,168,169]. International organizations have defined values, including PTDI, RfD, and CSF, for several of these chemicals [170,171]. Therefore, in chemical risk assessment studies concerning processing-induced contaminants, methods for carcinogenic and noncarcinogenic health risk assessment are used.

Food additives constitute a significant source of chemical exposure, occasionally raising public concern. Food additives, including preservatives, colorants, sweeteners, flavor enhancers, and stabilizers, are intentionally added to foods to extend their shelf life, improve appearance, or enhance flavor, thereby constituting a specific food chemical category. Before being approved for use, additives should undergo in-depth toxicological testing, with safety evaluations establishing specific limits for safe consumption. Thus, when used in accordance with good manufacturing practices (GMPs), the daily intake levels are anticipated to remain below the ADI, thereby presenting no risk to consumers. Scientific authorities, including the Joint FAO/WHO Expert Committee on Food Additives (JECFA) and EFSA, have assessed multiple additives and identified acceptable usage levels [172,173]. Nonetheless, certain additives remain widely debated. For example, nitrite and nitrate salts, frequently used as meat product preservatives, are recognized for their ability to form potent N-nitrosamine compounds, which are classified as carcinogenic, during cooking or within the stomach, despite their relatively low intrinsic toxicity [174]. The IARC has reported an association between processed meat consumption and colorectal cancer, with an aspect of this relationship attributed to the formation of nitrite-derived nitrosamines [175]. Potassium sorbate and sodium benzoate are broadly used preservatives in food products. Sorbate mainly prevents mold and yeast growth, whereas benzoate is more effective against bacteria. Both additives are affected by ADI limits of 3.70 and 0.07 mg/kg/day, respectively [176]. To guarantee consumer safety, legal regulation and regular oversight influence their utilization. Artificial sweeteners, including aspartame, have long been employed as sugar substitutes. The IARC has recently classified aspartame as Group 2B (possibly carcinogenic to humans) [177]; however, the JECFA reaffirmed that at current consumption levels (ADI = 40 mg/kg/day), aspartame does not pose a health risk [178]. This case emphasizes a crucial distinction between hazard identification, as performed by the IARC, and risk assessment, as conducted by the JECFA, highlighting the significant impact of exposure level and usage context on determining the actual risk. This case has also underscored the necessity of regularly revising scientific assessments of additives. Furthermore, certain synthetic food colorants, including tartrazine and Allura Red, have been associated with hyperactivity behaviors in children, leading to the introduction of warning labels and recommendations to limit their consumption [179,180]. Overall, the intake of approved food additives in a standard diet is considered within safe limits; additives associated with potential health risks have been either prohibited or are regulated under strict controls regarding their application. The case of food additives demonstrates effective risk assessment and management, as regulatory frameworks are regularly revised on the basis of emerging scientific evidence, and ADI values are reassessed as needed. In the food additive risk assessment, as most additives do not exhibit carcinogenic potential, noncarcinogenic health risk assessment methods are primarily utilized.

Industrial by-products or environmental residues constitute another group of chemicals unintentionally entering the food chain. In this category, polychlorinated biphenyls (PCBs), dioxins (PCDD/Fs), phthalate esters (PAEs), and perfluoroalkyl substances (PFAs) are recognized as persistent organic pollutants. Dioxins and PCBs, arising from industrial combustion processes, are toxic compounds that can remain in the environment for prolonged periods and accumulate in the food chain, especially in high-fat animal-derived foods [181,182,183]. Consequently, to mitigate dioxin and PCB exposure, the European Union (EU) has implemented a series of preventive measures throughout the food chain, from animal feed to final food products [184,185]. PAEs, used as plasticizers, are contaminant compounds migrating into food mainly from packaging materials, processing equipment, or environmental contamination [186]. They are frequently noted in increased concentrations in fat-rich foods, dairy products, infant formulas, and packaged beverages [187,188,189]. The endocrine-disrupting properties of PAEs have raised concerns regarding their potential adverse effects on reproductive health, hormonal balance, and development [190,191]. Therefore, the presence of phthalates in foods is closely monitored within exposure assessment frameworks, and national and international regulations restrict their use [192]. Certain PFA compounds can penetrate into the food sources, particularly fish, milk, and eggs, via environmental pathways and accumulate in human blood, potentially resulting in adverse health outcomes [193,194]. This finding prompted an immediate regulatory reaction; as of 2023, the EU established legal maximum limits for PFAs in selected foods [195]. Furthermore, several industrial chemicals, including melamine contaminants and mineral oil mixtures, occasionally raise food safety concerns [196]. Risk management in this field underscores regulating contamination sources via food packaging standards and GMPs, alongside establishing routine monitoring programs and early warning systems for safeguarding consumer health.

During production or storage, contaminants from packaging materials may migrate into foods. PAEs function as plasticizers to improve the flexibility of plastics, whereas BPA serves as a monomer in polycarbonate plastics and the inner coatings of canned food containers. These contaminants may leach into foods in trace amounts and exert estrogenic effects in the body, thereby disrupting hormonal balance [131,197,198]. Restrictions on BPA use in food-contact materials have recently increased, causing the extensive availability of “BPA-free” products [192].

The diversity of dietary exposure pathways is illustrated with the aforementioned chemicals. Individuals who maintain a balanced and varied diet are generally not exposed to high levels of any chemical. Conversely, unbalanced eating patterns, including excessive fish consumption or dependence on one food source, alongside irregular food production and control practices, including the availability of foods with high pesticide residues, may boost potential risks. Consequently, food safety authorities promote diverse dietary habits and regulate the levels of the aforementioned chemicals in foods through legislative measures. Ultimately, identifying and managing exposure sources are as critical as risk assessment in safeguarding public health.

## 5. International Guidelines and Risk Assessment Approaches

Major international authorities and guidance documents are crucial in evaluating foodborne chemical risks as they ensure the standardization of scientific methodologies and the comparability of results globally.

### 5.1. Codex Alimentarius and FAO/WHO

The Codex Alimentarius is an international commission co-managed by the FAO and WHO, involving member countries, focused on the development of food standards, guidelines, and codes of practice. The principles of risk analysis established by Codex require that the management of foodborne hazards is grounded in scientific risk assessment, which member states adopt and incorporate into their national legislations. In this framework, Codex has formed expert committees for designated groups of chemicals. The Joint FAO/WHO Meeting on Pesticide Residues (JMPR) formulates recommendations for ADI, ARfD, and MRLs concerning pesticide residues. Concurrently, JECFA performs risk assessments related to food additives, contaminants, and veterinary drug residues. The Codex decisions are based on the scientific outputs of committees such as JECFA and JMPR, reflecting an international consensus on food safety. The Environmental Health Criteria (EHC 240) series published by FAO/WHO serves as a foundational reference for risk assessment methodologies. FAO/WHO offers a comprehensive framework for evaluating chemical risks in foods, encompassing hazard identification through to risk characterization. Codex contributes to national risk assessment efforts by providing practical tools, including the Dietary Exposure Assessment Model, which enables countries to conduct assessments using their own data. Codex standards are acknowledged by the World Trade Organization (WTO), functioning as a common framework for international food trade and facilitating the harmonization of food safety regulations globally. The Codex Alimentarius system constitutes the backbone of the international food safety approach based on scientific risk assessment [199,200,201,202].

### 5.2. EFSA

EFSA is the organization responsible for performing scientific risk assessments related to food and feed safety in the European Union. The structure comprises specialized scientific panels and committees, each concentrating on distinct areas such as pesticides (PRIA Panel), food additives (ANS Panel), and contaminants (CONTAM Panel), which routinely publish scientific opinions and risk assessment reports. EFSA’s activities cover various subjects, including pesticides, food additives, contaminants, and packaging chemicals. The agency adheres to a transparent and evidence-based methodology that is consistent with the principles of the Codex Alimentarius. EFSA has pioneered the regulatory application of the MOE concept, officially adopting it in 2005 for the evaluation of genotoxic and carcinogenic substances. The institution was a pioneer in advancing the TTC concept and has produced comprehensive risk assessment reports on various contaminants, including acrylamide, dioxins, and heavy metals. EFSA has established guidelines for cumulative risk assessment that focus on the combined risk of pesticides with similar toxic modes of action. In Europe, risk management is carried out by the European Commission and Member States, informed by the scientific advice of EFSA, with the precautionary principle significantly influencing decision-making processes. Currently, EFSA is acknowledged as the gold standard in scientific food risk assessment globally, establishing benchmarks for methodological rigor, transparency, and harmonization in food safety science [20,46,203,204,205,206].

### 5.3. US EPA and FDA

The Environmental Protection Agency (EPA) in the United States is tasked with the risk assessment of pesticides and environmental contaminants, while the Food and Drug Administration (FDA) regulates food additives and food contact materials. The EPA oversees the Integrated Risk Information System (IRIS), a publicly available database that combines essential toxicological reference values for chemicals, such as RFD and cancer slope factors. The risk assessment framework developed by EPA adheres to the classical four-step model and is notably recognized for its quantitative characterization of cancer risk. The agency has introduced significant concepts into the scientific literature, including the application (uncertainty) factor, which is a foundational element of contemporary risk assessment methodology. The FDA performs thorough risk assessments prior to designating a substance as “Generally Recognized as Safe (GRAS)” or as an approved food additive. The FDA utilizes migration modeling and applies exemption criteria for food contact materials with low exposure levels, serving as regulatory tools within the risk assessment process [207,208,209,210].

### 5.4. IARC

IARC, a specialized agency of the WHO, is dedicated to cancer research and is particularly recognized for its Monograph Programme. This programme assesses the carcinogenic potential of various chemical, physical, and biological agents, along with the conditions of exposure. The IARC Monographs evaluate numerous substances and exposure types, categorizing them into Groups 1, 2A, 2B, and 3 according to the strength of the scientific evidence. In this classification system, Group 1 agents are classified as “carcinogenic to humans,” signifying sufficient evidence of carcinogenicity in humans. Group 2A and Group 2B indicate agents that are classified as “probably carcinogenic to humans” and “possibly carcinogenic to humans,” respectively. Group 3 refers to agents that are “not classifiable as to their carcinogenicity to humans” [211,212]. The evaluations of IARC concentrate exclusively on hazard identification, specifically assessing whether an agent has the potential to cause cancer, rather than determining the exposure levels at which it may present a risk. Risk assessors utilize these classifications to evaluate the magnitude of risk linked to actual exposure levels. Consequently, IARC classifications function as guidance tools for risk prioritization; for instance, if a substance classified as Group 1 is identified in foods, regulatory authorities may implement measures to reduce or eliminate exposure. IARC serves as a crucial archive of scientific knowledge, conducting systematic reviews of epidemiological and mechanistic evidence beyond simple classification. In the area of food safety, IARC has released monographs addressing mycotoxins, processed meat, and pesticides, among various other sources of exposure. The findings of the agency are used to guide national cancer control programs and global cancer prevention strategies, thereby emphasizing the incorporation of scientific evidence into public health policy.

### 5.5. Others

National and regional authorities, including Japan’s Food Safety Commission (FSCJ), Food Standards Australia New Zealand (FSANZ), and China’s National Center for Food Safety Risk Assessment (CFSA), have established important frameworks and methodologies for chemical risk assessment. In the European Union, additional scientific organizations, including the European Chemicals Agency (ECHA) and the Scientific Committee on Consumer Safety (SCCS), are crucial for the assessment of chemicals not related to pesticides, such as food contact materials and cosmetic ingredients. Under the REACH Regulation, ECHA evaluates the overall human exposure risks of chemicals, considering dietary intake as one of the exposure pathways. The Organization for Economic Co-operation and Development (OECD) advances risk assessment science through the development of international testing guidelines and tools, including the QSAR Toolbox, which aids in predicting chemical toxicity based on structural properties. Institutions such as the Joint Research Centre (JRC) play a crucial role in the development of new approach methodologies (NAMs), which encompass non-animal testing strategies. These methodologies seek to modernize and enhance risk assessment practices in accordance with the principles of sustainability and ethical science [213,214,215,216].

International guidelines aim to establish interoperability and consistency in chemical risk assessment. Guidance documents published by organizations such as EFSA, WHO, and FAO are acknowledged and used as reference standards by risk assessors across the world. Despite operating within different regulatory contexts, major international and national authorities share several core principles in chemical risk assessment. All systems—including Codex Alimentarius, EFSA, EPA/FDA, IARC, and regional bodies such as FSCJ, FSANZ, CFSA, ECHA, and SCCS—base their evaluations on scientific evidence, structured methodologies, transparent documentation, and expert committee review. Most frameworks follow the classical sequence of hazard identification, hazard characterization, exposure assessment, and risk characterization, and all aim to protect public health through consistent, reproducible, and internationally aligned procedures. Thereby, scientific parameters, including ADI values, established in one country generally align with those defined in other countries, favoring uniformity among regulatory authorities. However, national socioeconomic contexts and risk perceptions can result in variations in risk acceptance criteria. For instance, in the EU, the precautionary principle may inhibit the approval of a pesticide in cases of insufficient data, while other countries may issue conditional approvals based on the evidence available.

At the same time, notable differences distinguish these systems. Codex provides globally harmonized standards informed by JMPR and JECFA assessments, whereas EFSA applies similar principles within the EU but places stronger emphasis on transparency, cumulative risk assessment, and the use of tools such as MOE and TTC. In the United States, EPA focuses on quantitative cancer-risk estimation and environmental contaminants, while FDA oversees food additives and food-contact materials through GRAS decisions and migration models. IARC is limited to hazard identification and does not quantify risk. Regional authorities such as FSCJ, FSANZ, and CFSA adapt these approaches to their national contexts, while EU bodies like ECHA and SCCS assess broader chemical categories under REACH. Complemented by OECD test guidelines and emerging NAMs, these systems collectively reflect shared scientific foundations but differ in regulatory priorities within global food safety risk assessment. Despite these differences, a scientifically unified methodology offers significant benefits for global food trade. By employing a single set of scientific criteria to assess food safety, the potential for double standards is reduced, thereby enhancing mutual trust and regulatory consistency across regions.

## 6. Current Discussions and Challenges

**Figure 2:**
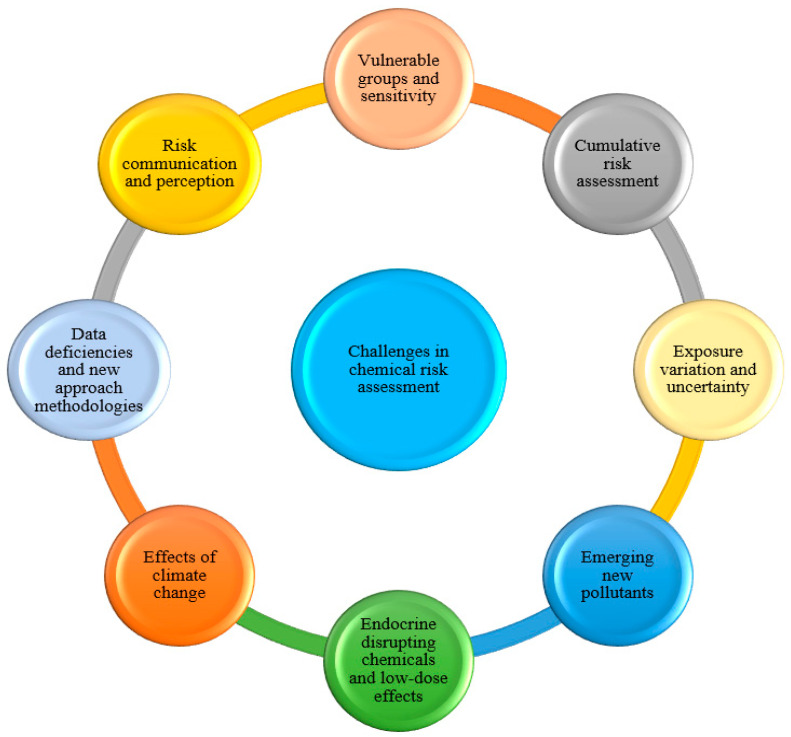
Challenges in chemical risk assessment.

Dietary chemical risk assessment is dynamic, constantly evolving in alignment with advancing scientific evidence and shifting exposure patterns. Several significant discussions and challenges have recently emerged in this area (Figure 2).

### 6.1. Sensitive Groups and Vulnerability

Compared with the general population, certain sensitive subpopulations, including infants, children, pregnant women, older adults, and individuals with compromised immune systems, may demonstrate increased vulnerability to chemicals. For instance, owing to their rapid growth and developmental phases, children exhibit heightened susceptibility to neurotoxic and endocrine-disrupting chemicals (EDCs). Owing to their higher food consumption relative to body weight and distinct metabolic capacities, children may experience a greater effective dose from an equivalent exposure level. In pregnant women, chemicals can pass from mother to fetus through the placenta, exposing the fetus to potential harm. Consequently, the EFSA and other regulatory agencies specifically consider these populations in risk assessments, frequently setting distinct ADI values or implementing additional safety margins when necessary. Protecting susceptible populations constitutes a core principle of risk management. For example, maximum contaminant levels established for infant formulas are substantially lower than those applicable to general foods, whereas fish consumption guidelines are more restrictive for pregnant women. The long-term health consequences of early life exposures are currently debated. Recent studies have indicated that chemical exposure during fetal or infant stages may cause negative outcomes in later life, including obesity and fertility disorders. This finding underscores the significance of a life course perspective and the need to focus on critical windows of susceptibility in risk assessment. Ultimately, risk assessors should evaluate risks not for the “average individual” but for the most sensitive members of the population, thereby ensuring adequate protection for all societal segments.

### 6.2. Cumulative Risk Assessment

Conventional risk assessment has mainly focused on individual chemicals; however, in reality, humans are concurrently exposed to various chemicals at low doses. The combined exposure, frequently called the “cocktail effect,” poses a significant challenge for regulators. For example, different pesticide residues in cereal products and plasticizers transferring from food packaging materials may interact to generate cumulative effects. In toxicology, chemical mixture complexity results in risk assessments typically assuming an additive effect, where doses of chemicals with similar mechanisms of action are summed to estimate the overall risk. Nevertheless, interactions among chemicals may either be synergistic or antagonistic; consequently, scientific research has significantly focused on mixture toxicity. EFSA conducted two comprehensive pilot studies to assess potential health risks from cumulative exposure to pesticides. These studies classified active substances with similar toxic mechanisms of action into Cumulative Assessment Groups (CAGs) and applied quantitative cumulative risk models, particularly for neurotoxic effects and effects on thyroid function [217,218]. Pilot results showed that cumulative exposure in all age groups did not exceed established reference values and remained within consumer safety limits. The US EPA conducted a comprehensive cumulative risk assessment, determining that organophosphate pesticides pose a significant cumulative risk due to their common toxic mechanism, acetylcholinesterase inhibition. This assessment modeled total organophosphate exposure by age group, combining food, drinking water, environmental contact, and indoor exposures. Exposure distributions were quantitatively estimated from national dietary surveys, environmental measurements, and drinking water concentrations; the total risk was calculated using a proportional risk characterization based on the RfD. The findings indicated that total organophosphate exposure was approaching critical thresholds in some sensitive subgroups [219]. In a preliminary cumulative risk assessment of di-(2-ethylhexyl) phthalate (DEHP) and dibutyl phthalate (DBP), intrauterine exposure was quantified in cord blood from 50 postpartum women, with mean concentrations of 99.9 µg/L for DEHP and 24.7 µg/L for DBP. Embryotoxicity testing in the human embryonic stem cell test yielded BMDL of 29.9 and 0.99 µg/mL for DEHP and DBP, respectively, and both phthalates were shown to inhibit embryonic development via the PPAR/PTEN/Akt signalling pathway. Based on these exposure levels and toxicity reference points, the cumulative risk for pregnant women with high exposure was judged to warrant particular concern [220]. Recent reviews have suggested that the diet of average European consumer comprises multiple potentially genotoxic or carcinogenic contaminants concurrently, with the combined risk from these mixtures potentially surpassing that of individual compounds [221,222,223]. This finding challenges the conventional risk assessment paradigm focused on one substance. In response, initiatives in Europe, such as the EuroMix Project, have been developing models and tools for evaluating the risks associated with multi-chemical dietary exposures. Cumulative risk analysis frequently employs indicators, including the HI and combined MOE. Regulators in the field of pesticides have initiated the calculation of cumulative ARfD and cumulative ADI values for chemical groups demonstrating a common mechanism of toxicity, such as organophosphates. Regulatory agencies are preparing to systematically integrate cumulative risk assessment outcomes into decision-making processes, underscoring the significance of the cocktail effect in real-world scenarios. The integration of dose–response relationships for various substances, the consideration of correlated exposures, and the modeling of chemical interactions remain significant challenges. Despite these complexities, scientific progress persists, and future risk management strategies seek to more effectively address the combined risks posed by co-occurring dietary chemicals.

### 6.3. Exposure Variability and Uncertainty

The dietary consumption of a chemical can significantly vary across individuals and over time. Differences in dietary habits mainly cause interindividual variability; for instance, a vegetarian and a meat-based eater will exhibit varying exposure levels to specific veterinary drug residues. Geographical and cultural variations significantly impact arsenic exposure from rice, with Asian populations demonstrating higher levels than their Western counterparts. Risk assessments have frequently analyzed upper percentiles of exposure distributions to account for variability, such as determining exposure for the 95th percentile consumer. Extreme dietary groups, including populations who predominantly consume fish, are independently evaluated as they may experience significantly elevated dioxin exposure compared with the general population. Temporal variation constitutes another aspect of variability. Individual diets vary daily; however, long-term average intake is the critical measure for chronic risk assessment. National dietary surveys have generally covered a consumption period of only 1–2 days or 1 week; therefore, statistical modeling is essential for estimating habitual intake levels over a 1-year period. To achieve this objective, “usual intake” models are employed to yield continuous consumption distributions. The EFSA advocates for evaluating advanced statistical methods, including the NCI model, to enhance the prediction of long-term exposure from short-term dietary data, thereby refining the precision of chronic exposure estimates. Conversely, each phase of risk assessment presents with uncertainty, including uncertainties in chemical concentration measurements, sampling errors in dietary surveys, and reporting biases in self-reported food intake data. Recent risk assessment reports have increasingly emphasized the significance of quantitative and qualitative characterizations of uncertainty, utilizing analyses (e.g., Monte Carlo simulations) for estimating 95% confidence intervals. Furthermore, to provide a more comprehensive perspective, scenario analyses comparing worst-case and typical exposure conditions are frequently utilized. All these efforts aim to provide risk managers a more informative and transparent risk characterization. However, fully capturing the variability of exposure remains nearly impossible. Consequently, to ensure that the actual risk is not undervalued, conservative assumptions are frequently applied, while also preserving a sufficient margin of safety in dietary chemical risk assessment.

### 6.4. EDCs and Low-Dose Effects

EDCs are substances that can interfere with the hormonal system, potentially disrupting vital physiological functions, including growth, reproduction, and metabolism. A key characteristic of EDCs is their capacity to display non-monotonic dose–response relationships, contradicting the conventional toxicological principle that asserts “the dose makes the poison.” In other words, at low doses, some endocrine disruptors can generate biological effects that are not evident at higher doses, frequently demonstrating U-shaped or inverted U-shaped response curves. For example, animal studies have shown that BPA can induce behavioral changes in mice at low doses; however, these effects are not observed at higher exposure levels [224]. These results complicate risk assessment, as conventional methods, based on high-dose animal studies and established threshold values, may insufficiently account for subtle low-dose effects. In a study of adult male and female CD-1 mice, exposure from conception until 12–14 weeks of age to BPA (4 to 40,000 µg/kg/day) or 17α-ethinyl estradiol (0.02 to 2 µg/kg/day) produced dose- and sex-specific alterations in spleen microstructure and immunomodulatory function, indicating that BPA can impact immune system development even at low doses [225]. Recent research has proposed ten ‘key characteristics’ of endocrine-disrupting chemicals, highlighting that EDCs may affect hormone receptors, alter hormone synthesis, transport or signaling, and thereby provoke diverse adverse health outcomes including reproductive dysfunction, metabolic disorders or cancer [226]. Endocrine disruptors have varied and intricate biological mechanisms, making the concept of a “safe dose” still a subject of ongoing scientific debate. Exposure during critical developmental periods and its long-term health outcomes, including prenatal exposure and its potential to increase disease risk later in life, are currently being thoroughly investigated. A joint report by the WHO and the United Nations Environment Program highlighted that endocrine disruptors pose a significant global public health risk, calling for global cooperation on this topic [227].

### 6.5. Emerging Pollutants

“Emerging contaminants” are chemicals or groups of chemicals that have been recently detected in environmental or food systems, are not routinely monitored, and may pose potential risks to human health or ecosystems due to limited toxicological or exposure data [228]. Several newly identified or emerging chemicals have recently entered the food chain, becoming an increased focus in risk assessment. Microplastics, defined as plastic particles smaller than 5 mm, have been detected in various food items, including seafood, drinking water, milk, honey, and salt [229,230,231,232,233,234]. Current studies have suggested that most microplastics are excreted without being absorbed by the human digestive system, and no definitive evidence of direct negative health impacts has been established [235,236]. However, microplastics may function as carriers for various substances, including monomers, additives, or adsorbed environmental chemicals, thereby introducing further uncertainty regarding their potential long-term health effects [237,238]. Owing to their persistence in the environment and continuous accumulation, the most suitable strategy for managing potential future risks is a precautionary approach. Nanotechnology-based materials, including nanomaterials in food packaging and residues from nano-fertilizers, entering the food supply constitute another emerging area of concern. In 2021, the EFSA released revised guidelines regarding the risk assessment of nanomaterials in food and feed, highlighting that a reduction in particle size to the nanoscale can significantly alter toxicokinetic and toxicological properties [239,240]. In this context, the adaptation of conventional testing protocols and the application of suitable characterization techniques are essential challenges in accurately assessing risks associated with nanomaterial exposure in the food chain.

### 6.6. Effects of Climate Change

Global climate change is reshaping the food safety risk profile. Increased temperature and humidity levels elevate the risk of mycotoxin contamination in agricultural crops, leading to the appearance of toxins that were previously rare in specific geographical regions. Similarly, warming sea temperatures may facilitate the proliferation of marine biotoxin-producing algae, causing increased toxin risks in seafood. Moreover, climate change may modify pesticide usage patterns, potentially resulting in the increased application of different chemical agents for managing changing pest populations. Therefore, the comprehension of secondary effects of climate change on chemical exposures represents a significant uncertainty in future risk assessment. In this context, modeling the potential scenarios to implement the preventive strategies is required.

### 6.7. Data Gaps and New Approach Methodologies (NAMs)

Owing to the wide variety of chemicals in the food environment, obtaining comprehensive toxicological data for every compound is practically impossible. To address this limitation, NAMs (e.g., Threshold of Toxicological Concern [TTC], Structure–Activity Relationships [SAR/QSARs] and read-across techniques [read-across from similar chemicals]) have become crucial instruments for risk assessors. Particularly for the numerous trace-level components migrating from food packaging materials, the TTC approach sets a conservative dose threshold based on specific structural classes (e.g., derived from the most toxic known chemicals) and assumes that exposures below this threshold indicate a negligible risk. The authorities, including the EFSA and FDA, apply these principles by excluding low-exposure chemicals from comprehensive evaluations, thereby directing resources toward higher-risk substances. However, the practical application of these approaches differs across regions: high-income countries generally possess advanced analytical capacity to support NAM-based evaluations, whereas low- and middle-income settings often face data gaps and limited monitoring infrastructure, which can restrict the reliable use of these models. Nonetheless, the application of NAMs involves uncertainties and challenges in acceptance, as these methods are frequently not considered established or reliable compared with conventional animal testing approaches. To enhance animal welfare and resource efficiency, regulatory authorities advocate for NAM use; however, they exercise caution in integrating these methods into decision-making processes. Recently, artificial intelligence and machine learning have significantly transformed predictive toxicology and large-scale data analysis. Although these technologies potentially improve the speed and comprehensiveness of risk assessment, debates continue regarding the transparency and accuracy of their outputs. Such disparities in technological capacity further widen the gap between countries in implementing AI-supported or data-intensive NAM tools.

### 6.8. Risk Communication and Perception

Effective risk communication is key to making risk assessment work in public health. Food-associated chemical risks can occasionally result in either excessive concern or apathy within media and public perception. When the IARC classified aspartame as a possible carcinogen, headlines, including “Is sweetener actually poison?” emerged, causing public confusion. Here, differentiating between “hazard” and “risk” and highlighting the significance of exposure level become essential for risk assessors and authorities. In the case of aspartame, the WHO officials aim to clarify that no safety concern associated with typical daily consumption levels of aspartame exist. Pesticide residues, food additives, and processing contaminants have also presented similar communication challenges. The primary goal of risk communication is to bridge the communication gap between scientists and the public. Moreover, in the current social media landscape, addressing misinformation and ensuring the reach of credible scientific information to the public are significant challenges.

Effective risk communication serves as a critical bridge between scientific assessment and public policy. Regulatory agencies such as EFSA, FDA, and WHO increasingly employ structured strategies to translate complex exposure-assessment outcomes into clear, actionable consumer guidance. These strategies include tiered messaging, where simplified consumer summaries accompany technical scientific opinions; transparency tools such as open-access databases and exposure dashboards; and coordinated multi-stakeholder communication to ensure consistent messaging across institutions. Such approaches enable policymakers to issue targeted dietary recommendations, provide context for acceptable risk levels, and counter misinformation. By integrating these communication frameworks, risk-assessment findings are more effectively transformed into practical guidance that supports informed consumer choices and strengthens public trust in food safety systems.

Therefore, ongoing discussions and new challenges make risk assessment evolving. The integration of mixture toxicity evaluation, interindividual sensitivity differences, early identification of emerging risks, integration of data science, and effective risk communication will shape the future direction of this discipline. A comprehensive, proactive, and transparent approach to risk assessment is warranted to address these challenges.

## 7. Future Perspectives

The assessment of carcinogenic and non-carcinogenic health risks from dietary chemical exposure is a core component of modern food safety science and requires a multidisciplinary approach. As outlined in this review, protecting human health against foodborne chemicals depends on a comprehensive framework that combines toxicological principles, robust exposure assessment methods, international standards, and emerging scientific developments.

Although current risk-assessment methodologies rely on strong scientific principles, continuous refinement is needed. Emerging priorities include evaluating cumulative chemical effects, protecting vulnerable populations, and understanding climate- and environment-driven changes in contamination patterns. In response, international food safety authorities are increasing cooperation, and collaboration among EFSA, WHO/FAO, and IARC is promoting greater scientific harmonization. Translating scientific assessments into policy action remains essential for maintaining a safe food supply, and this process is increasingly standardized and regularly updated in line with scientific progress.

Evaluations suggest that the general population is usually exposed to the majority of foodborne chemicals at levels considered to be safe. This has been accomplished via the adoption of good agricultural and manufacturing practices, regulatory maximum limits, and effective monitoring systems. Nonetheless, some remaining areas concerning specific subgroups or special situations still need to be improved. From a food safety management perspective, it is crucial that the results of risk assessments be translated into risk-management decisions—such as withdrawal of risky products and the establishment of new regulations—which requires strong communication between risk assessors and decision-makers. First and foremost, a preventive approach (proactive risk management) should be adopted.

Future projections may indicate several key trends in the area of dietary chemical exposure. First, a shift toward a more holistic risk assessment approach exists, highlighting the evaluation of the overall risk–benefit balance of the total diet instead of focusing on individual chemicals. Second, integrating technological advancements into risk assessment is anticipated to rise. Advanced high-sensitivity analytical techniques will facilitate identifying trace chemicals in foods that were previously undetectable, whereas biosensors and real-time monitoring systems will enable individual-level exposure tracking. The increasing accessibility of biomonitoring data will improve risk assessment by directly linking it to internal exposure indicators. Third, the risk profiles of emerging agricultural and food technologies designed for tackling climate and environmental challenges, including biopesticides and renewable packaging, will increasingly be incorporated into the assessment agenda. Potential risks arise with the introduction of new technology, requiring an ongoing process of learning and adaptation in risk assessment. Fourth, heightened consumer awareness is anticipated, emphasizing the need for effective risk communication. Consumers are increasingly seeking information regarding the chemical composition of their food and are turning toward clean-label products. This demand encourages the industry to implement safer alternatives and drives the scientific community to assess the safety of these alternatives. Consequently, regulatory authorities are compelled to communicate scientific findings more clearly and develop policy measures that align with evolving consumer expectations. Fifth, risk management initiatives undertaken by individual countries are inadequate owing to the transnational nature of contaminants; therefore, enhancing global collaboration is crucial. Finally, specific policy areas, including the combat against food fraud, the control of environmental pollution, and the promotion of good agricultural practices (e.g., integrated pest management), are essential for mitigating chemical risks at their source.

Numerous scientific questions remain unresolved. In particular, the long-term health effects of low-dose chemical mixtures and their cumulative impacts are still not fully understood, and the debate on whether endocrine-disrupting chemicals exert effects below established thresholds continues. Significant challenges also persist in linking biomarker data with epidemiological outcomes to establish causality, underscoring the need for more integrated research. Although artificial intelligence and big-data tools offer promising opportunities for improving dietary pattern analysis and exposure prediction, their potential remains largely untapped. Additionally, the early identification and assessment of emerging hazards will become increasingly important as new risks enter the research agenda. Overall, sustained investment in research and interdisciplinary collaboration remains essential.

Effective risk management relies on monitoring. Implementing extensive surveillance programs, including TDS, on an international scale enables nations to gain a deeper understanding of their exposure profiles. To integrate new analytical techniques for screening a wider array of compounds, national food monitoring programs require ongoing updates. Traceability throughout the food chain facilitates the rapid identification of contamination sources when difficulties arise. Furthermore, biomonitoring programs are essential for identifying priority exposures relevant to public health. The monitoring data are essential for sustaining the risk assessment cycle and evidence-based policymaking.

## 8. Conclusions

In conclusion, dietary chemical exposure management necessitates scientific rigor, foresight, transparency, and collaboration. This review highlights the central message that effective risk assessment must integrate toxicological principles, robust exposure methodologies, international standards, and emerging innovations to safeguard public health. Achieving effective management relies on a multidisciplinary approach aligning with scientific progress, effectively handling uncertainties, and engaging various stakeholders. Progressions in this process will cause a decreased incidence of foodborne diseases and an enhanced public trust in food safety. Prospectively, minimizing chemical risks is a key step to establish a safer and more sustainable food system. In this context, risk assessors should identify hazards and adopt a proactive approach by providing solution-oriented recommendations. Furthermore, within the One Health framework, the interconnections between the environment and human health in risk assessment will be emphasized, considering that several foodborne chemicals originate from environmental pollution.

From the perspective of current challenges, several issues remain particularly critical. First, key scientific uncertainties persist—including the long-term effects of low-dose chemical mixtures, unresolved questions surrounding endocrine-disrupting chemicals at subthreshold doses, and the difficulty of linking biomarker data with epidemiological outcomes. Second, the emergence of climate-driven and environmentally driven shifts in contamination patterns continues to complicate exposure assessment. Third, ensuring effective risk communication and maintaining public confidence remain ongoing priorities, especially amid increasing misinformation. These challenges underline the continued need for strong scientific foundations, data quality, and coordinated risk governance.

Looking ahead, this review points to three future priorities that will shape research and risk assessment practices. First, the refinement of cumulative and mixture toxicity assessments will be essential to better characterize combined exposures. Second, the integration of advanced tools—including high-resolution analytical methods, biosensors, biomonitoring, and data-driven technologies such as artificial intelligence—will significantly enhance exposure modelling and early detection of emerging hazards. Third, strengthening internationally harmonized frameworks and expanding global data-sharing initiatives will help ensure consistent, science-based policy actions in the face of transnational chemical risks.

To establish a safe, diverse, and sustainable food system is the common and ultimate goal behind all these initiatives. Chemical risk assessment is a crucial tool for attaining this goal. Utilizing science-based and internationally coordinated risk assessment practices can effectively manage hidden hazards in food, thereby promoting a healthier nutritional environment for future generations. Adopting balanced and varied eating habits acts as, in essence, an individual-level risk management strategy, inhibiting excessive exposure from a single source while maximizing nutrient benefits. Consequently, an effective reduction in dietary chemical risks is possible through a multilayered awareness and collective action, ranging from individuals to society.

## Figures and Tables

**Table 1:** The most frequently used concepts and their significance in hazard characterization.

Abbreviation/ Concept	Definition and Importance
NOAEL (No-Observed-Adverse-Effect Level)	The highest experimental dose level at which no adverse effects are observed. It is determined in animal studies and is generally used as the Point of Departure (PoD) in risk assessment. The NOAEL serves as a reference point for establishing safe intake levels for humans [17].
LOAEL (Lowest-Observed-Adverse-Effect Level)	The lowest experimental dose at which an adverse effect is first observed, indicated by a statistically significant change. In the absence of a NOAEL in a study, the LOAEL value can serve as a Point of Departure (PoD); however, the application of additional uncertainty factors is necessary in this context [17].
ADI (Acceptable Daily Intake)/TDI (Tolerable Daily Intake)	The amount of a substance that, based on current knowledge, is considered to pose no appreciable health risk when consumed daily over a lifetime (usually expressed in milligrams per kilogram of body weight). The calculation is typically performed by dividing the NOAEL or BMDL from animal studies by relevant safety (uncertainty) factors. The term ADI refers to substances intentionally added into foods, including food additives, pesticide residues, and veterinary drug residues, whereas TDI is typically used for substances that occur unintentionally, such as industrial contaminants [17].
ARfDs (Acute Reference Dose)	The maximum dose considered acceptable for health after acute exposure (less than 24 h). It is generally assessed for substances with potential acute toxicity, as exemplified by pesticides. If the intake of an individual remains below this threshold during a single meal or day, it is considered that there is no risk of acute poisoning. ARfD value is expressed in mg/kg and is derived from acute toxicity studies [17].
BMD/BMDL (Benchmark Dose/Lower Confidence Limit)	The BMD is derived from statistical modeling of the experimental dose–response curve and is typically defined as the dose expected to result in a 10% incidence of an adverse effect within the population (e.g., tumor formation). The Benchmark Dose Lower Confidence Limit (BMDL) denotes the lower confidence limit, generally set at 95%, for this dosage. The BMD approach serves as an alternative to the NOAEL method, especially in studies characterized by strong dose–response data. The BMDL value can serve as a Point of Departure (PoD) and be divided by appropriate safety factors. In contemporary risk assessment, the BMD method is favored over the NOAEL as it more accurately accounts for experimental uncertainty and employs the complete dataset [17].
MOE (Margin of Exposure)	The Margin of Exposure (MOE) is a ratio used specifically in the risk characterization of genotoxic and carcinogenic substances. The calculation involves comparing an experimentally derived reference dose, typically the BMDL or LOAEL from animal studies, with the actual human exposure. A higher MOE value indicates a reduced risk linked to the existing level of exposure to that substance. For genotoxic carcinogens, EFSA has established a practical guidance criterion: an MOE value of 10,000 or higher, calculated from the BMDL_10_ derived from animal data, signifies a low level of concern for public health. The MOE functions as a quantitative measure, offering risk managers insight into the potential need for additional action [17,20].
TTC (Threshold of Toxicological Concern)	The TTC is a preliminary screening concept designed for numerous substances lacking adequate toxicological data. Daily intake levels regarded as unlikely to cause harm have been established for particular groups of chemical structures based on current knowledge. The TTC value for a structurally simple organic compound considered inert may be 1.5 µg/kg body weight per day. If actual exposure is below this threshold, it can be concluded that comprehensive toxicity testing and risk assessment for that substance are unnecessary. The TTC approach was established as a practical method for prioritizing substances from a vast array of compounds, including flavoring agents and migration products. Of course, the TTC does not constitute a definitive assurance of safety; instead, it reflects an assumption of a negligible risk threshold. Exceeding the TTC value necessitates further toxicological assessment [17].

**Table 2:** Environmental and agricultural contaminants and their key properties in dietary chemical exposures.

Contaminants/ Main Food Sources	Sources of Contamination	Potential Health Effects
Pesticides: Fruits, vegetables, cereal grain products, and other pesticide-treated agricultural products	Pesticide contamination in foods mainly results from the use of insecticides, herbicides, and fungicides in agricultural production. These chemicals can reach foods through leaves, fruits, soil, or water, and may also enter the food chain via polluted irrigation water, residual soil contamination, or treatments applied during storage and transport. In animal-derived foods, residues generally occur indirectly through contaminated feed [56,57,58,59].	The health effects of pesticides vary depending on the specific compound, exposure duration, and individual susceptibility. Acute exposure may cause nausea, vomiting, dizziness, respiratory distress, skin or eye irritation, neurological symptoms, and, in severe cases, organ failure [60,61]. Chronic low-dose exposure is linked to impaired immune function, endocrine disruption, neurological and reproductive problems, and fetal developmental effects [62,63,64,65,66]. Certain pesticide classes, including organophosphates and carbamates, have also been identified by international agencies as potential or probable human carcinogens [19].
Potentially toxic metals: Cereal grains and grain products, fruits and vegetables, seafoods, particularly large fish species and shellfish, milk and dairy products, meat and meat products, and other foods contaminated with potentially toxic metals	Contamination sources of potentially toxic metals in food primarily arise from environmental and technological processes. The application of fertilizers, pesticides, and irrigation water in agriculture can result in the migration of metals into soil and subsequently into plant-based foods. Industrial activities and environmental pollution lead to the accumulation of mercury and arsenic, especially in seafood, whereas feed and water contamination serve as significant sources in animal-derived foods. Moreover, the processing, storage, and packaging stages can facilitate the migration of metals, including aluminum and lead, into food products [67,68,69,70].	Potentially toxic metals accumulate in the body due to their non-biodegradable nature and can adversely affect multiple organs. Lead and mercury primarily damage the central nervous system and are associated with learning difficulties, cognitive impairment, and behavioral disorders in children [71,72,73]. Cadmium can reduce bone mineral density and cause irreversible kidney damage [74,75]. Arsenic exposure is linked to skin lesions, cardiovascular problems, hepatotoxicity, and several cancers, including skin, lung, and bladder cancer [76,77]. Long-term aluminum exposure has been associated with neurodegenerative disorders, particularly Alzheimer’s disease [78,79]. Chromium may impair kidney function, while cobalt and nickel can trigger immune reactions and respiratory irritation [80,81,82]. These effects vary depending on age, physiological status, and exposure duration, with children, pregnant women, and immunocompromised individuals being the most vulnerable.
Mycotoxins: Cereal grains and grain products (corn, wheat, barley, rice), legumes and oilseeds (peanut, hazelnut, walnut, soy), dried fruits (dried fig, apricot, raisins), spices (red pepper, black pepper), coffee and coffee products, milk and dairy products, and other foods contaminated with mycotoxins.	Mycotoxin contamination arises from both primary sources, such as the field, and secondary sources, including post-harvest, storage, and processing stages. Primary contamination takes place during the crop growth period in the field, influenced by factors including high humidity, temperature variations, insect damage, and inadequate agricultural practices, which facilitate the proliferation of molds such as Aspergillus, Fusarium, and Penicillium. Secondary contamination occurs post-harvest due to insufficient drying, inadequate ventilation, humid and warm storage conditions, as well as during processing and transportation. The occurrence of mycotoxins in food results directly from environmental conditions and practices during production and storage [83,84,85,86].	Mycotoxins can exert both acute and chronic toxic effects and are well known for their carcinogenic, mutagenic, teratogenic, and immunotoxic properties [87,88]. Aflatoxins—especially aflatoxin B_1_—are highly hepatotoxic and may cause acute liver failure as well as increase the risk of liver cancer with long-term exposure [89,90]. Ochratoxin A accumulates in the kidneys, leading to nephrotoxicity, impaired renal function, and potential carcinogenicity [91,92]. Fumonisins affect the nervous system and have been associated with congenital abnormalities such as neural tube defects [93,94]. Trichothecenes inhibit protein synthesis, resulting in immune suppression and gastrointestinal symptoms including nausea, vomiting, and bleeding disorders [95,96]. Zearalenone, due to its estrogenic activity, disrupts hormonal balance and may cause reproductive disorders [97,98]. The severity of these effects depends on exposure dose, duration, and individual susceptibility, with children, pregnant women, and immunocompromised individuals being the most vulnerable. Overall, mycotoxins pose a substantial public health concern, contributing to both non-carcinogenic risks such as nephrotoxicity and immunotoxicity, and carcinogenic risks, particularly hepatocarcinogenesis.

**Table 3:** Processing, additive, and packaging contaminants and their key properties in dietary chemical exposures.

Contaminants/ Main Food Sources	Sources of Contamination	Potential Health Effects
Process contaminants HMF: Honey, fruit juices, dried fruits, coffee, UHT milk, and bread, etc. Acrylamide: French fries, potato chips, coffee, biscuits, crackers, breakfast cereals, and bread, etc. HCAs: Meat, poultry, fish and processed meat products, etc. Chloropropanols: Refined vegetable oils, margarine, frying oils, soy sauce, etc. PAHs: Meat, and fish, as well as roasted coffee, cocoa, nuts, certain vegetable oils, and cereals, etc.	HMF, acrylamide, HCAs, chloropropanols, and PAHs are widely formed in foods during thermal processing, cooking, manufacturing, or storage. HMF appears when sugar-containing foods are exposed to high temperatures or prolonged storage, while acrylamide develops during frying, baking, and roasting via the Maillard reaction. HCAs form in protein-rich foods such as meat and fish cooked at high temperatures. Chloropropanols are generated mainly during the processing of refined vegetable oils, soy sauce, and other fat-based products. PAHs commonly arise through smoking, charring, and roasting methods [116,117,118,119].	High intakes of HMF may exert cytotoxic effects [120]. Acrylamide is primarily neurotoxic and has been classified by the IARC as probably carcinogenic to humans [19,121]. HCAs are mutagenic, capable of inducing DNA damage, and have been linked to increased risks of colon, pancreatic, and prostate cancers with long-term exposure [122,123,124]. Chloropropanols show nephrotoxic and reproductive toxicity, and some derivatives have been evaluated for their carcinogenic potential [19,125,126]. PAHs possess strong mutagenic and carcinogenic properties, with compounds such as benzo[a]pyrene clearly associated with cancer development in humans [19,127,128].
Food additives Nitrite and nitrate: processed meat products (e.g., soudjouk, sausages, salami, hot dogs, and ham), and some vegetables. Potassium sorbate and sodium benzoate: fruit juices, carbonated beverages, pickles, ketchup and other sauces, some canned products, jams, marmalades, and other foods in which they are used. Aspartame: sugar-free chewing gums, sweetener tablets, and other foods containing it.	Nitrite, nitrate, potassium sorbate, sodium benzoate, aspartame, and various other additives are intentionally incorporated into foods, each serving a specific technological function. Food additives are essential for ensuring the safety, stability, and sensory quality of food products [129,130,131].	High exposure to nitrites and nitrates may increase cancer risk due to the formation of methemoglobinemia and nitrosamines [132,133,134]. Although potassium sorbate is generally considered safe, excessive intake may trigger allergic reactions or gastrointestinal discomfort [135,136]. Sodium benzoate may also cause toxic effects at high levels or when combined with benzoic acid derivatives [137,138]. Aspartame is widely used as a low-calorie sweetener but poses risks for individuals with phenylketonuria and has been linked to headaches and neurological symptoms when consumed in excess [139,140]. Additionally, some colorants and emulsifiers have been associated with further health concerns [141].
Packaging and environmental contaminants PCBs: Fatty fish, milk and dairy products, meat and meat products, other contaminated foods. PCDD/Fs: fish and shellfish, fatty meat products, milk and dairy products, other contaminated foods. PAEs: Milk and dairy products, vegetable oils, processed foods, and other contaminated foods. PFAS: fish, seafood, and other contaminated foods. BPA: canned foods, beverages packaged in plastic bottles, and other processed foods.	PCBs, PCDD/Fs, PAEs, PFAS, and BPA contaminate food mainly via environmental pollution and migration from packaging materials. While PCBs and PCDD/Fs are primarily transferred to animal-derived foods through industrial emissions, combustion processes, and contaminated soil or water; PAEs migrate into food from plastic materials used as plasticizers. PFAS compounds are present in foods as a result of their application in food packaging coatings and their environmental durability. BPA, on the other hand, is a substantial source of contamination, as it transitions from the inner linings of canned foods, plastic bottles, and baby bottles into food and beverages [142,143,144,145,146,147,148].	PCBs, PCDD/Fs, PAEs, PFAS, and BPA are significant concerns due to their persistence and bioaccumulation. PCBs accumulate in the body and have been linked to immune suppression, neurodevelopmental issues, endocrine disruption, liver damage, and cancer [149,150,151]. PCDD/Fs are highly toxic and associated with skin lesions, immune dysfunction, and reproductive and developmental problems [152,153,154]. PAEs act as endocrine disruptors and may affect reproductive health, sperm quality, hormonal balance, and fetal development, posing particular risks for infants and children [155,156,157]. PFAS compounds bioaccumulate and are associated with thyroid disorders, altered lipid metabolism, elevated liver enzymes, immune suppression, and higher risks of kidney and testicular cancers [158,159,160]. BPA, through its estrogen-mimicking activity, disrupts endocrine function and has been linked to obesity, insulin resistance, infertility, early puberty, cardiovascular disease, and hormone-related cancers [161,162,163].

## Data Availability

No new data were created or analyzed in this study. Data sharing is not applicable to this article.
